# Pre-sleep screen time and screen time addiction as shared determinants of poor sleep and obesity in adolescents aged 11–14 years in Scotland

**DOI:** 10.1186/s44263-025-00160-y

**Published:** 2025-05-07

**Authors:** Emma Louise Gale, Andrew James Williams, Joanne E. Cecil

**Affiliations:** 1 School of Medicine, University of St. Andrews, Scotland, UK; 2 School of Health in Social Sciences, University of Edinburgh, Scotland, UK

**Keywords:** Teenagers, Insomnia, Sleep disturbance, Screens, Mobile phones, Overweight, Adiposity

## Abstract

**Background:**

The overall quantity of screen time has been associated with short sleep duration and increasingly sedentary lifestyles, leading to adiposity. The aim of this research was to explore which components of screen time usage are shared determinants of poor sleep and higher adiposity in adolescents, using data from the Teen Sleep Well Study.

**Methods:**

A cross-sectional study of adolescents aged 11–14 years in Fife, Scotland was conducted. Sleep was measured objectively using the Actigraph GT3X-BT and subjectively using validated questionnaires. Adiposity was assessed using body fat percentage (BF%) and obesity was measured using body mass index percentile (BMIp). Four components of screen time were addressed using questionnaires: the timing of screen time, quantity of screen time, location of screen time, and screen time addiction. Descriptive statistics and statistical tests such as Pearson correlation tables, and adjusted regression analyses were used. Mediation analyses explored wellbeing as a factor in the association between screen time and sleep and obesity.

**Results:**

Sixty-two participants (33 female/29 male, mean age 12.2 ± 1.1 years, mean BMIp 60.3 ± 32.1) completed the study. Excessive screen time pre-sleep (30 min before sleep) and post-sleep (first 30 min after waking), excessive screen time on a weekend, and screen time addiction were shared determinants of higher adiposity, a later chronotype (evening-preference) and poor sleep outcomes: poor sleep habits, increased insomnia symptoms (IS) and increased sleep onset variability. Mediation analyses confirmed that adolescent wellbeing mediated the association between pre-sleep screen time and IS (36.3%) and BF% (21.9%), post-sleep screen time and IS (37.7%) and BF% (30.4%), videogaming addiction and IS (31.9%) and BF% (34.6%), social media addiction and IS (35.0%) and BF% (17.4%), mobile phone addiction and IS (34.0%) and BF% (10.6%), weekday screen time and IS (58.1%) and BF% (39.8%), and weekend screen time and IS (51.4%) and BF% (38.0%).

**Conclusions:**

These screen time behaviours, alongside wellbeing should be considered in multi-component health-promoting interventions aimed at improving adolescent sleep and reducing obesity risk. Future research should employ longitudinal designs to clarify the directionality of these associations and determine the effectiveness of interventions that target both screen time behaviours and wellbeing.

**Supplementary Information:**

The online version contains supplementary material available at 10.1186/s44263-025-00160-y.

## Background

The pervasive use of digital screens, addictive social media, apps and new digital technology has become an integral part of modern adolescence, significantly influencing health outcomes [1]. During the COVID-19 pandemic, there was a shift to online and remote school-working and socialisation causing a shift to adolescents increasingly using screens for everyday activities and becoming more dependent on digital devices [2–5]. Previous research has reported that excessive screen time among adolescents has a detrimental impact on both sleep and obesity [1, 6–9]. The rapid proliferation of smartphones, tablets, and computers has led to significant changes in sleep patterns, disrupting circadian rhythms and altering chronotypes [10, 11]. Prolonged screen exposure, particularly before bedtime, is associated with delayed sleep onset [12], insomnia symptoms [13], short sleep duration [14], and increased social jetlag [15]. These disruptions in sleep habits contribute to reduced physical activity and increased sedentary behaviour [16–18], behaviours identified as critical factors in the rising prevalence of obesity among youth [19–21]. However, cross-sectional, temporal, and longitudinal studies have produced inconsistent findings regarding the association between screen time, sleep, and obesity. While cross-sectional research frequently reports significant associations between screen use and poor sleep or increased adiposity, temporal and longitudinal studies have yielded more mixed results, suggesting that screen time alone may not be the sole driver of these outcomes [22]. Instead, these discrepancies suggest that more complex interactions may be at play, including the timing, location, and context of screen use, or the influence of additional factors such as mental wellbeing, lifestyle behaviours, and individual differences in sleep patterns. A recent systematic review highlighted that there is limited research into the timing, location, and context of screen time usage [6]. Identifying the specific components of habitual screen time that are most detrimental to health is crucial for designing effective health-promoting interventions [6]. Different aspects of screen use, such as the type of content consumed [23–25], the timing of usage [12, 26, 27], and screen time addiction [5, 28–31], could each have distinct impacts on adolescent health and the developing brain. For example, engaging in stimulating activities like gaming or social media use before bedtime can exacerbate insomnia symptoms [14, 32], whereas passive screen time, such as watching TV, has been shown to contribute more significantly to sedentary behaviour and obesity [33, 34]. Additionally, excessive late-night pre-sleep screen time can interfere with critical periods of brain development, affecting cognitive functions, emotional regulation, and mental health [9, 35]. By pinpointing these components, interventions can be tailored to address the most harmful habits.

While excessive screen time has been associated with poor sleep and increased adiposity, the mechanisms underlying these associations remain complex. Wellbeing, has been found to be associated with excessive screen time [36] and plays a role in the development of poor sleep [37]and obesity [38] cycles, operating bidirectionally. Adolescents with high screen exposure, particularly at night, often experience psychological distress, increased stress levels, and reduced overall wellbeing, which in turn may contribute to sleep disturbances [37] and unhealthy lifestyle behaviours that promote adiposity [38]. Rather than solely being an outcome of screen time use, wellbeing could be a key factor in the association between screen time, sleep, and obesity, potentially acting as a mediator. Understanding whether wellbeing mediates these associations is useful, as this might suggest that interventions focusing only on reducing screen time may be insufficient—instead, addressing mental health and wellbeing alongside screen time modification could be a more effective strategy for improving adolescent health outcomes.

Consequently, the research question addressed by this study was: are different components of problematic screen time usage shared determinants of poor sleep and higher adiposity in adolescents? The different components of screen time examined in this study included pre-sleep screen time (30 min prior to sleep), post-sleep screen time (first 30 min after waking), quantity of screen time, location of screen time, and screen time addiction. The research questions were addressed by conducting the Teen Sleep Well Study (TSWS), a cross-sectional study in Scotland, measuring sleep, adiposity, obesity, screen time and other behavioural variables. The study cohort consisted of 62 adolescents aged 11–14 years from North-East Fife, Scotland, recruited from the general community.

## Methodology

### Study design, recruitment, and procedure

**Fig. 1:**
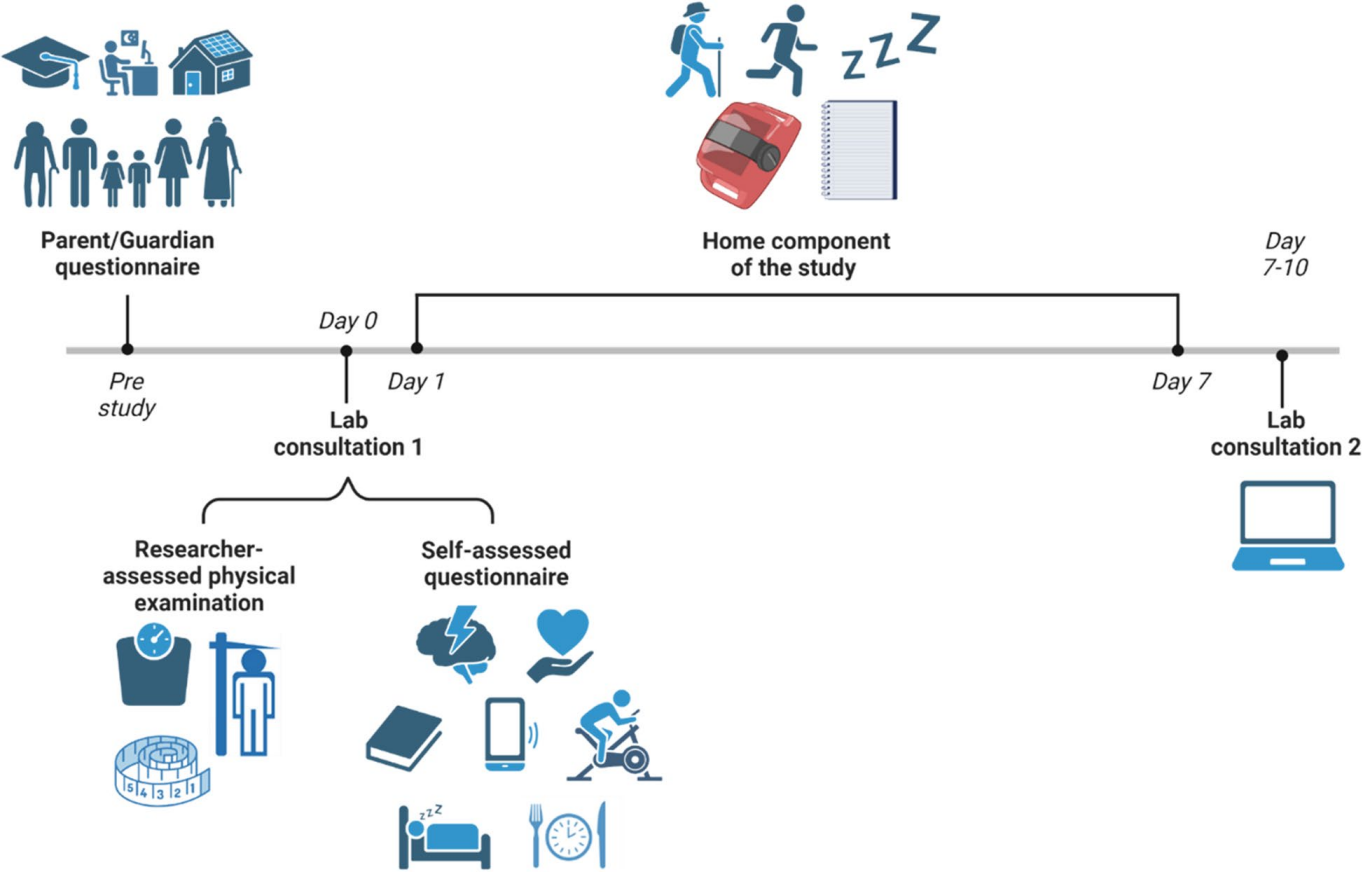
Cross-sectional primary research study protocol (TSWS), including pre-study caregiver consent, Consultation 1, Home component and Consultation 2 [39]

The Teen Sleep Well Study (TSWS), a cross-sectional quantitative study, was conducted, including a caregiver-assessed questionnaire, adolescent-assessed questionnaire, researcher-assessed objective anthropometry, and 7–10 days (including weekdays (structured) and weekends (unstructured) during the school term-time) of actigraphy in 11–14-year-olds (Fig. 1).

Adolescent participants (aged between 11 and 14 years) and their caregivers were recruited from Northeast Fife, a county in Scotland, via flyers at leisure centres, cafes, youth centres, transport centres, University memo adverts, and online Facebook community groups. Inclusion criteria for the study included (i) being 11–14 years at recruitment, (ii) enrolled at a school in Fife, (iii) informed consent obtained from the caregiver, and (iv) free of underlying health conditions and medication use. Exclusion criteria included (i) < 11 years or > 14 years, (ii) < 3 weekdays and < 1 weekend days of actigraphy data available for the adolescent participant, (iii) caregiver questionnaire, including consent, is absent, (iv) medication use, and (v) already having a sibling from the same household take part in the study. A G*Power calculation was completed to determine the desired study sample size. The recommended effect, power and error sizes for a regression analysis were used [40]. An *f*-test linear regression test was conducted (effect size *F*2 = 0.30, α = 0.05, power = 0.8, number of predictors = 8, primary outcomes = sleep duration and obesity, which suggested a sample of 59 would be sufficient for an actual power of 0.807.

### Research variables

#### Caregiver demographics

Caregivers were asked to describe their relationship to the adolescent, date of birth, gender, ethnicity, marital status, and postcode. If a second adult lived in the home, for example a spouse, partner, another guardian or grandparent, their date of birth, gender and ethnicity was also recorded. Caregivers were asked “what is your highest level of education” with answers and scores of “none or few school qualifications” (0), “secondary school leaver” (1), “sixth form/college or apprenticeship” (2), “university degree” (3) or “masters or postgraduate degree” (4). Caregivers were asked to report their current employment status and shift patterns. For example, “as part of your work, do you work night shifts?” and “as part of your work do you work shifts that finish late in the evening (after 11pm)?” with answers of “never” (0) “rarely” (1), “sometimes” (2), “often” (3), “all the time” (4) and “not applicable” (0). A higher score indicating more frequent late or night shifts. If another adult lived in the household, the same questions were asked of the second household member. Caregivers were asked for their height (cm or in feet and inches) and weight (kg or stones and pounds), and body mass index (BMI) (kg/m^2^) was then calculated.

#### Adolescent demographics

The adolescent’s date of birth, gender, ethnicity, and pubertal status were self-reported. Pubertal status was assessed using six questions from the Health Behaviour in School-aged Children (HBSC) study [41]. Adolescent participants answered either male- or female-specific questions regarding any pubertal developments or could opt not to answer either and move on to the next section of the questionnaire. Socioenvironmental status was derived using the Scottish Index of Multiple Deprivation (SIMD) [42] quintile data from the participant’s home postcode reported by the caregiver.

#### Anthropometry

The adolescent participant’s height (cm) and weight (kg) were measured three times by a trained researcher (EG), and a mean was calculated. Height was measured using a portable stadiometer (The Leicester Height Measure, Seca Ltd., Birmingham, UK) (0.1 cm precision) [43], with no shoes, and standing straight with the adolescent participant’s head facing forward [43], and a mean was then calculated. Adolescent weight (kg) was assessed using Tanita body composition scales (TBF-300M, Tanita Corporation, Tokyo, Japan) (0.1 kg precision) [44].

Adolescent adiposity (body fat percentage) was measured using bioelectrical impedance (Tanita body composition scales, TBF-300M, Tanita Corporation, Tokyo, Japan) (0.1 kg precision) [44], via bioelectrical impedance [45] and a mean of the three readings was then calculated [44]. This method provides reliable estimates of body composition in a quick, low cost, non-invasive manner that increases compliance [46]. Adolescent participants were asked to remove shoes and socks, wear light clothing and empty their pockets [44]. Adiposity status groups (under-fat, healthy-fat, over-fat and obese) were determined by UK age- and gender-specific validated standard cut-offs [47].

#### Chronotype and sleep

*Chronotype* was assessed using the 19-item Morningness Eveningness Questionnaire (MEQ) [48]. All 19-items were multiple choice, with scores being allocated to each answer and combined for a final score. A final score of ≤ 41 indicated an “evening type”, 42–58 indicated an “intermediate type” and ≥ 59 indicated a “morning type”. The MEQ has been validated in adolescents [49] and has been used in multiple sleep studies with adolescents [50, 51]. Cronbach's α coefficients have varied across validity studies (α > 0.80) in different countries, for example New Zealand recorded reliability of α = 0.83 [52] and Slovenia recorded a reliability of α = 0.86 [53] in a normative adult sample.

*Sleep habits* were assessed using an adapted eight-domain and 33-item children’s sleep habit questionnaire (CSHQ) [54]. The original questionnaire has been validated and used in children and adolescents [54, 55] with the Cronbach’s α reliability coefficient ranging from 0.68 in the community and 0.78 in clinical paediatric populations [54]. The questionnaire was adapted to allow adolescent participants to self-report rather than caregiver-report. The questionnaire assesses bedtime resistance, sleep onset latency, sleep duration, sleep anxiety, night wakings, parasomnias, sleep-disordered breathing, and daytime sleepiness. Questions were asked on a Likert scale, for example “Adolescent goes to bed at the same time at night”. Answers and scores were “always (7 days)” (4), “usually (5–6 days a week)” (3), “sometimes (2–4 days a week)” (2), “rarely (1 day a week)” (1) and “never” (0). A higher score indicated better and more regular sleep habits.

*Insomnia symptoms* were assessed using the 7-item insomnia severity index (ISI) [56]. The seven questions were scored on a Likert scale 0–4. For example, “How satisfied/dissatisfied are you with your current sleep pattern?” “very satisfied” (0), “satisfied” (1), “moderately satisfied” (2), “dissatisfied” (3) and “very dissatisfied” (4). The scores of the seven items are combined, and a total score of 0–7 indicated no clinically significant insomnia, 8–14 is subthreshold insomnia, 15–21 indicated moderate clinical insomnia, and 22–28 indicated severe clinical insomnia [56]. The ISI was initially validated in 17–82 years [56] but has since been validated in the adolescent population with a Cronbach’s α reliability coefficient of 0.83 [57].

*Sleep duration and sleep onset variability* was assessed by actigraphy [58], with the adolescent wearing an Actigraph GT3X-BT (ActiGraph LLC, Pensacola, FL, USA) [59] for 7–10 days. The Actigraph GT3X-BT has been validated for capturing sleep variables (including sleep timing, efficiency and awakenings) in children and adolescents (including those with obesity) [58] against the gold standard, polysomnography (90.2% accuracy, 95.7% sensitivity, and 62% specificity) [59]. In this study, for the data to be valid, ≥ 3 weekday nights and ≥ 1 weekend night was required. The sleep variables were detected and formulated using the Cole-Kripke algorithm [60], and a consensus sleep diary was used to corroborate sleep onset [61]. Sleep onset variability was defined as the standard deviation of an individual’s sleep onset.

#### Wellbeing (quality of life)

*Quality of life (QoL)* was self-assessed using KIDSCREEN-27 [62]. The KIDSCREEN-27 is a validated 27-item version of KIDSCREEN-52 and had a Cronbach’s alpha reliability coefficient of above 0.78 in all domains [63]. Participants answered questions on the five domains: physical wellbeing (5 items), psychological wellbeing (7 items), autonomy and caregiver relation (7 items), peers and social support (4 items) and school environment (4 items). Items were scored on a Likert scale 1–5, for example “Have your caregiver(s) treated you fairly?” was answered with “never” (5), “seldom” (4), “quite often” (3), “very often” (2) or “always” (1). A total for each domain and a total score for all 27 items is then calculated, a higher score indicated a poorer QoL.

#### Screen time

##### Quantity of screen time

Self-assessed using two domains from the validated SCREENS questionnaire (SCREENS-Q): screen media environment and children’s screen use [64]. Participants were asked to report how many different types of electronic devices were in the household and how often the adolescent participant had access to them on a weekday and weekend. Adolescents were also asked about whether the caregiver set screen time guidelines, how often the adolescent participant used screen time guidelines and how long the adolescent participant used screen media on a weekday and weekend.

##### Timing of screen time

Pre-sleep and post-sleep screen time was assessed by four questions: (1) “How many days do you use your phone on a weekday in the first 30 min after waking up?” and (2) “How many days do you use your phone on a weekday in the last 30 min before bed?”, and the answers were “none”, “1–2 days a week”, “3–4 days a week” or “5 days a week”. The same questions were asked for the weekend with answers of “none”, “one day at the weekend” or “both days at the weekend”.

##### Location of screen time

Whether the adolescent used screen time in bed or in the bedroom was assessed using three questions reported by the adolescent: (1) Do you use screens whilst in bed? (2) Do you use screens in your bedroom? (3) Do you use your phone as an alarm?

#### Screen time addiction

Social media addiction (SMA-Q), video gaming addiction (VGA-Q) and mobile phone addiction (MPA-Q), were self-assessed using three domains from the Adolescent Brain and Cognitive Development study questionnaire (ABCD): VGA-Q (six items), SMA-Q (six items) and MPA-Q (8 items) [65]. THE VGA-Q and SMA-Q, both consisted of a Likert scale with answers “Never” (0), “Very rarely” (1), “Rarely” (2), “Sometimes” (3), “Often” (4) and “Very often” (5) [65]. The MPA-Q also consisted of a Likert scale with answers “Strongly disagree” (1), “Disagree” (2), “Somewhat disagree” (3), “Neither disagree, nor agree” (4), “Somewhat agree” (5), “Agree” (6), “Strongly disagree” (7) [65]. A The MPA-Q was validated [66] and further used [67] in adolescents prior to the ABCD study use. The SMA-Q and VGA-Q were first used and validated by the ABCD study and were designed based off the validated Bergen Facebook Addiction Scale [68]. Higher scores for each individual questionnaire, SMA-Q, VGA-Q, and MPA-Q indicated the individual was increasingly addicted to the social media, videogaming and mobile phone use, respectively.

### Statistical analysis

The data were extracted and analysed using IBM SPSS Statistics for Windows, Version 28.0 (IBM Corp., Armonk, NY, USA) and R (R Foundation for Statistical Computing, Vienna, Austria). Missing data were assessed using the Little’s Missing Completely At Random (MCAR) test [69]. No missing data were identified following assessment, and therefore no imputation or deletion methods were required. Frequency diagrams and descriptive tables were used to demonstrate sample characteristics of the adolescent participants and caregivers. *T*-tests were used to assess the difference between adiposity status groups (under-fat and healthy-fat versus over-fat and obese). Pearson correlations and descriptive statistics were used to identify correlations between screen time and sleep, obesity, and adiposity in the adolescent participants. Block-wise regression analysis was used to examine which screen time variables were independently associated with the sleep and adiposity outcomes. Adjusted variables were selected based on associations identified in unadjusted analyses or have been previously identified as shared determinants of poor sleep and obesity in adolescents [6]. Block 1 and 2 adjusted for demographics of the adolescents and caregivers, respectively, that were associated with poor sleep and obesity in the unadjusted analyses (Table 1). Block 3 adjusted for wellbeing (QoL) as an indicator of wellbeing (Table 1). Block 4 of the regression analysis included the screen time variable (Table 1). Significant associations from the regression analyses were identified using a ± 10% difference (β ≤  −0.1 or ≥ 0.1). Based on the regression analysis, the sleep and obesity variables with the strongest association with screen time were used to conduct mediation analyses using the laavan package on R [70]. The mediation analysis was conducted to examine whether wellbeing (QoL) mediated the association between screen time and sleep and adiposity.

**Table 1:** Adjusted variables in the blockwise regression analyses of screen time on adiposity and sleep outcomes in adolescent participants

Block number	Adjusted for
1	Gender Ethnicity
2	Maternal employment status Maternal night shifts Maternal late shifts Maternal BMI
3	Quality of life
4	One screen time habit (i) Timing of screen time (last 30 min and first 30 min of the day) (ii) Quantity of screen time (on a weekday and weekend) (iii) Location of screen time (use phone in bed, in the bedroom and as an alarm) (iv) Addictive tendencies of screen time (social media, videogaming, and mobile phone addiction)

*Key: BMI *body mass index, *min *minutes

## Results

### Sample description

Sixty-six participants were initially recruited to the study, 62 completed the study and were included in the study analysis. The adolescent participants included in the analysis were from North-East Fife, Scotland, including 29 males and 33 females, with a mean age of 12.2 ± 1.1 years (Table 2).

**Table 2:** Participant characteristics: adolescent and caregiver demographics

Demographic variables		Total			
		*N*	%	Mean	SD
Adolescent gender	Male	29	46.8		
	Female	33	53.2		
Adolescent age (year)		62		12.2	1.1
Adolescent ethnic minority	White British	50	80.6		
	Other	12	19.4		
Adolescent body development (pubertal)	Male	29		7.9	3.3
	Female	33		10.6	3.6
Adolescent body fat percentage (%)	Male	29		18.9	12.6
	Female	33		25.2	9.6
Adolescent body mass index percentile (BMIp)	Male	29		62.3	32.4
	Female	33		58.3	32.3
Adolescent and caregiver SES (SIMD)	SIMD rank	62		5209.5	1259.2
	Quintile 1	0	0.0		
	Quintile 2	0	0.0		
	Quintile 3	10	16.1	4.3	0.8
	Quintile 4	35	56.5		
	Quintile 5	17	27.4		
Highest level of maternal education	≤ 16 years	4	6.5		
	16–18 years	13	21.0		
	Undergraduate	18	29.0		
	Postgraduate	27	43.5		
Maternal employment status	Full-time	34	54.9		
	Part-time	21	33.9		
	Student	2	3.2		
	Retired/homemaker	3	4.8		
	Other	2	3.2		
Frequency of maternal night shifts	Never/NA	54	87.1		
	Rarely	0	0		
	Sometimes	1	1.6		
	Often	7	11.3		
Frequency of maternal late shifts	Never/NA	47	75.8		
	Rarely	4	6.5		
	Sometimes	5	8.1		
	Often	6	9.7		
Maternal age (years)		62		44.46	7.23
Maternal BMI (kg/m^2^)		62		24.62	4.70
Number of caregiver household figures		62		1.76	0.43

Key: *BMIp* body mass index percentile, *kg* kilograms, *m* metres, *NA* not applicable, *SD* standard deviation, *SIMD* Scottish Index of Multiple Deprivation (2020)

Mean body fat percentage was 22.3 ± 11.5% and mean BMIp 60.3 ± 32.1. There were significant differences between adiposity groups (under-fat/healthy-fat: UF/HF (*n* = 40) and over-fat/obese: OF/OB (*n* = 22)) across all sleep outcomes (Table 3), and screen time dimensions (Table 4). Sleep characteristics reported in adolescents in the over-fat and obese adiposity status include later chronotype, poorer sleep habits, more severe insomnia symptoms, later sleep onset and longer sleep onset latency compared with those in the under-fat/healthy-fat group (Table 3). Significant differences between body fat status groups were reported in the timing of screen time (screen time use in the first 30 min of the day and the last 30 min of the day), quantity of screen time (hours of screen time on a weekday and weekend), location of screen time (phone use in bed, having phone in the bedroom overnight and using phone as an alarm) and screen time addiction (videogaming, social media, and mobile phone) (Table 4).

**Table 3:** Participant characteristics: adolescent sleep by adiposity status

Sleep outcome		UF/HF (*n* = 40)		OF/OB (*n* = 22)		Total (*n* = 60)	
		Mean	SD	Mean	SD	Mean	SD
Morningness-eveningness questionnaire		51.15	9.37	30.91	10.51	43.97	13.77
Child sleep habits questionnaire		43.10	10.74	59.82	10.39	49.03	13.27
Insomnia severity index		6.13	5.77	23.09	4.68	12.15	9.79
Sleep onset (time)	WD	22:38	00:55	00:29	00:45	23:17	01:14
	WE	23:23	01:11	01:54	01:11	00:17	01:41
Sleep onset latency (mins)	WD	27.52	21.38	51.05	14.27	35.87	22.16
	WE	28.83	26.16	44.45	25.93	34.37	26.94
Sleep duration (mins)	WD	464.86	50.25	347.52	75.71	423.22	82.41
	WE	487.10	61.90	527.81	91.31	501.55	75.54

*Key*: *HF* healthy-fat, *OB* obese, *OF* over-fat, *mins* minutes, *WD* weekday, *WE* weekend

**Table 4:** Adolescent screen time by adiposity status

Screen time variable		UF/HF				OF/OB				Total		
		*N*	%	Mean	SD	*N*	%	Mean	SD	*N*	Mean	SD
Timing of screen time												
First 30 min of the day (WD)	None	19	30.6			1	1.6			20		
	1–2 days a week	12	19.4			1	1.6			13		
	3–4 days a week	6	9.7			2	3.2			8		
	5 days a week	3	4.8			18	29.0			21		
Last 30 min of the day (WD)	None	19	30.6			0	0.0			19		
	1–2 days a week	11	17.7			1	1.6			12		
	3–4 days a week	8	12.9			4	6.5			12		
	5 days a week	2	3.2			17	27.4			19		
First 30 min of the day (WE)	None	17	27.4			0	0.0			17		
	1 day	17	27.4			3	4.8			20		
	Both days	6	9.7			19	30.6			25		
Last 30 min of the day (WD)	None	17	27.4			0	0.0			17		
	1 day	21	33.9			5	8.1			26		
	Both days	2	3.2			17	27.4			19		
Screen time addiction												
Videogaming		40		9.30	4.69	22		25.55	7.28	62	15.06	9.68
Social media		40		11.27	8.03	22		28.09	8.21	62	17.24	11.41
Mobile phone		40		23.63	11.31	22		46.45	8.66	62	31.73	15.13
Quantity of screen time												
Quantity of screen time	WD	40		7.45	4.27	22		14.95	2.87	62	10.11	5.26
	WE	40		9.17	4.70	22		17.27	2.71	62	12.05	5.65
	Var	40		1.80	1.90	22		2.32	1.29	62	1.98	1.71
Location of screen time												
Use of phone in bed	Yes	19	30.6			22	35.5			41		
	No	21	33.9			0	0.0			22		
Phone in the bedroom overnight	Yes	21	33.9			22	35.5			43		
	No	19	30.6			0	0.0			19		
Phone as an alarm	Yes	11	17.7			22	35.5			33		
	No	29	46.8			0	0.0			29		

*Key*: *HF* healthy-fat, *N* number, *OB* obese, *OF* over-fat, *min* minutes, *SD* standard deviation, *WD* weekday, *WE* weekend, *var* variability

### Unadjusted associations

#### Screen time and adiposity

Higher adiposity (higher body fat percentage, larger waist circumference, larger hip circumference, larger waist-to-hip ratio, larger waist-to-height ratio) and a higher BMIp were meaningfully associated with pre-sleep and post-sleep screen time (weekday and weekend), a higher screen time quantity (weekday and weekend), screen time addiction (videogaming, social media and mobile phone) and use of phone in bed, in the bedroom overnight and as an alarm (Table 4).

#### Screen time and sleep

Poorer sleep outcomes (a later chronotype, poorer sleep habits, more severe insomnia symptoms, later sleep onset on a weekday and weekend, a higher sleep onset variability and longer sleep onset latency on a weekday and weekend) were meaningfully associated with pre-sleep and post-sleep screen time (weekday and weekend), a higher screen time quantity weekday and weekend), screen time addiction (videogaming, social media and mobile phone) and use of phone in bed, in the bedroom overnight and as an alarm (Additional file 1).

### Adjusted associations

Model summaries for blocks 1, 2, 3, and 4 (i-iv) have been reported in Table 5.

**Table 5:** Model summaries of the blockwise regression of screen time behaviours on adiposity, obesity, and sleep outcomes in adolescents

	Model 1 *(adjusted for demographics of the adolescent participants)*				Model 2 *(adjusted for model 1* + *demographics of the caregivers)*				Model 3 *(adjusted for model 2* + *wellbeing of the adolescent participants)*				Model 4 *(adjusted for model 3* + *one screen time variable (i) timing (ii) quantity (%), (iii) location or (iv) addiction of the adolescent participants*					
	*R*^2^	AdjR^2^	*F*	*p*	*R*^2^	AdjR^2^	*F*	*p*	*R*^2^	AdjR^2^	*F*	*p*	*R*^2^		AdjR^2^	ΔAdjR^2^	*F*	*p*
Dependent variable																		
Body fat percentage	0.099	0.068	3.226	0.047	0.290	0.198	3.158	0.007	0.695	0.649	15.090	< .001	(i)	0.830	0.796	*0.147*	24.866	< .001
													(ii)	0.743	0.692	*0.034*	14.714	< .001
													(iii)	0.804	0.761	*0.096*	18.656	< .001
													(iv)	0.832	0.795	*0.137*	22.551	< .001
Body mass index percentile	0.064	0.032	1.996	0.145	0.426	0.351	5.626	< .001	0.453	0.369	5.378	< .001	(i)	0.466	0.359	*−0.010*	4.361	< .001
													(ii)	0.488	0.385	*0.016*	4.757	< .001
													(iii)	0.466	0.346	*−0.023*	3.886	< .001
													(iv)	0.571	0.475	*0.106*	5.927	< .001
Chronotype	0.055	0.023	1.720	0.188	0.305	0.214	3.378	0.005	0.791	0.759	25.014	< .001	(i)	0.859	0.818	*0.059*	25.204	< .001
													(ii)	0.820	0.784	*0.025*	25.014	< .001
													(iii)	0.808	0.765	*0.006*	19.099	< .001
													(iv)	0.822	0.782	*0.023*	20.940	< .001
Sleep habits	0.041	0.008	1.251	0.294	0.249	0.151	2.555	0.024	0.574	0.509	8.912	< .001	(i)	0.607	0.531	*0.022*	7.893	< .001
													(ii)	0.618	0.543	*0.034*	8.236	< .001
													(iii)	0.635	0.555	*0.046*	7.919	< .001
													(iv)	0.604	0.517	*0.008*	6.925	< .001
Insomnia symptoms	0.027	−0.006	0.831	0.441	0.307	0.217	3.410	0.004	0.726	0.685	17.561	< .001	(i)	0.848	0.819	*0.134*	28.514	< .001
													(ii)	0.785	0.743	*0.058*	18.632	< .001
													(iii)	0.822	0.783	*0.098*	20.981	< .001
													(iv)	0.882	0.857	*0.172*	34.112	< .001
Sleep onset variability	0.052	0.020	1.627	0.205	0.355	0.271	4.240	< .001	0.729	0.688	17.837	< .001	(i)	0.801	0.762	*0.074*	20.525	< .001
													(ii)	0.801	0.762	*0.074*	20.496	< .001
													(iii)	0.770	0.720	*0.032*	15.230	< .001
													(iv)	0.785	0.738	*0.050*	16.632	< .001

*Key: AdjR*^*2*^AdjR^2^*, DV *dependent variable*, ΔAdjR*^*2*^Change of AdjR^2^

#### Screen time and body fat percentage

Frequent pre-sleep screen time usage (β = 2.862, Confidence interval (CI) (95%) = 1.389, 4.336), frequent use of a phone in bed (β = 9.452, CI (95%) = 3.644, 15.259), videogaming addiction (β = 0.333, CI (95%) = 0.055, 0.611) and social media addiction (β = 0.372, CI (95%) = 0.046, 0.697) were significantly associated with higher body fat percentage in adolescents (Additional file 1). The independent coefficients of the quantity of screen time were not significantly associated with body fat percentage (Additional file 1).

#### Screen time and body mass index percentile

Videogaming addiction (β = 1.027, CI (95%) = 0.223, 2.278) and social media addiction (β = 1.575, CI (95%) = 0.108, 3.043) were significantly associated with higher BMIp in adolescents (Additional file 1). The independent coefficients of the timing, quantity and location of screen time were not significantly associated with BMIp (Additional file 1).

#### Screen time and chronotype

Frequent pre-sleep screen time (β = 0.558, CI (95%) = 0.346, 1.230), post-sleep screen time (β =  − 1.851, CI (95%) =  − 3.617, − 0.084) and a higher quantity of screen time on a weekend (β =  − 1.098, CI (95%) =  − 2.117, − 0.077) were significantly associated with a later chronotype in adolescents (Additional file 1). The independent coefficients of the location of screen time and screen time addiction were not significantly associated with chronotype in adolescents (Additional file 1).

#### Screen time and sleep habits

A higher quantity of screen time on a weekend (β = 1.588, CI (95%) = 0.171, 3.005) and keeping the phone in the bedroom overnight (compared with not) (β =  − 10.938, CI (95%) =  − 19.892, − 1.984) were significantly associated with poorer sleep habits in adolescents (Additional file 1). The independent coefficients of the timing of screen time and screen time addiction were not significantly associated with sleep habits in adolescents (Additional file 1).

#### Screen time and insomnia symptoms

Frequent post-sleep screen time usage (β = 1.644, CI (95%) = 0.470, 2.818), frequent pre-sleep screen time usage (β = 1.634, CI (95%) = 0.446, 2.822), frequent use of a phone as an alarm (compared with not) (β = 8.137, CI (95%) = 3.861, 12.413), videogaming addiction (β = 0.428, CI (95%) = 0.229, 0.627), and social media addiction (β = 0.273, CI (95%) = 0.040, 0.506) were significantly associated with more severe insomnia symptoms in adolescents (Additional file 1). The independent coefficients of the quantity of screen time were not significantly associated with insomnia symptoms in adolescents (Additional file 1).

#### Screen time and sleep onset variability

Frequent pre-sleep screen time usage (β = 867.766, CI (95%) = 313.039, 1422.494) was significantly associated with a larger sleep onset variability in adolescents (Additional file 1). The independent coefficients of the quantity and location of screen time and screen time addiction were not significantly associated with sleep onset variability in adolescents (Additional file 1).

### Mediation analysis: wellbeing and association between screen time, adiposity, and sleep

#### Pre-sleep screen time and body fat percentage and insomnia symptoms

Quality of life partially mediated 21.9% of the association between pre-sleep screen time and body fat percentage, and 36.3% of the association between pre-sleep screen time and insomnia symptoms (Table 6).

**Table 6:** Mediation analysis of quality of life on the association between the timing of screen time and body fat percentage and insomnia symptoms

	Estimate (β)	SE	Confidence interval (95%)		% mediation
			Lower	Upper	
Pre-sleep screen time and body fat percentage					
Indirect	1.109	0.409	0.375	1.950	21.9
Direct	3.957	0.551	2.890	5.064	78.1
Total	5.066	0.421	4.222	5.894	100.0
Pre-sleep screen time and insomnia symptoms					
Indirect	1.572	0.486	0.839	2.757	36.3
Direct	2.754	0.568	1.461	3.695	63.7
Total	4.326	0.261	3.789	4.808	100.0
Post sleep screen time and body fat percentage					
Indirect	1.377	0.470	0.512	2.361	30.4
Direct	3.159	0.567	2.060	4.318	69.6
Total	4.536	0.433	3.659	5.389	100.0
Post-sleep screen time and insomnia symptoms					
Indirect	1.542	0.480	0.7227	2.587	37.7
Direct	2.545	0.599	1.232	3.601	62.3
Total	4.087	0.289	3.449	4.602	100.0

#### Post-sleep screen time and body fat percentage and insomnia symptoms

Quality of life partially mediated 30.4% of the association between post-sleep screen time and body fat percentage, and 37.7% of the association between post-sleep screen time and insomnia symptoms (Table 6).

#### Videogaming addiction and body fat percentage and insomnia symptoms

Quality of life partially mediated 34.6% of the association between videogaming addiction and body fat percentage, and 36.3% of the association between videogaming addiction and insomnia symptoms (Table 7).

**Table 7:** Mediation analysis of quality of life on the association between screen time addiction and body fat percentage and insomnia symptoms

	Estimate (β)	SE	Confidence interval (95%)		% mediation
			Lower	Upper	
Videogaming addiction and body fat percentage					
Indirect	0.320	0.108	0.111	0.552	34.6
Direct	0.604	0.148	0.321	0.906	65.4
Total	0.925	0.095	0.751	1.117	100.0
Videogaming addiction and insomnia symptoms					
Indirect	0.282	0.084	0.130	0.462	31.9
Direct	0.601	0.089	0.418	0.776	68.1
Total	0.883	0.044	0.806	0.979	100.0
Social media addiction and body fat percentage					
Indirect	0.151	0.077	0.003	0.311	17.4
Direct	0.717	0.113	0.503	0.954	82.6
Total	0.868	0.077	0.727	1.019	100.0
Social media addiction and insomnia symptoms					
Indirect	0.257	0.070	0.134	0.412	35.0
Direct	0.477	0.098	0.274	0.661	65.0
Total	0.734	0.059	0.615	0.839	100.0
Mobile phone addiction and body fat percentage					
Indirect	0.069	0.082	− .102	0.231	10.6
Direct	0.581	0.110	0.376	0.800	89.4
Total	0.650	0.056	0.544	0.755	100.0
Mobile phone addiction and insomnia symptoms					
Indirect	0.188	0.079	0.029	0.342	34.0
Direct	0.365	0.098	0.161	0.556	66.0
Total	0.553	0.039	0.474	0.626	100.0

#### Social media addiction and body fat percentage and insomnia symptoms

Quality of life partially mediated 17.4% of the association between social media addiction and body fat percentage, and 35.0% of the association between social media addiction and insomnia symptoms (Table 7).

#### Mobile phone addiction and body fat percentage and insomnia symptoms

Quality of life partially mediated 10.6% of the association between mobile phone addiction and body fat percentage, and 34.0% of the association between mobile phone addiction and insomnia symptoms (Table 7).

#### Weekday screen time and body fat percentage and insomnia symptoms

Quality of life partially mediated 39.8% of the association between weekday screen time and body fat percentage, and 58.1% of the association between weekday screen time and insomnia symptoms (Table 8).

**Table 8:** Mediation analysis of quality of life on the association between the quantity of screen time and body fat percentage and insomnia symptoms

	Estimate (β)	SE	Confidence interval (95%)		% mediation
			Lower	Upper	
Weekday screen time and body fat percentage					
Indirect	0.659	0.198	0.280	1.071	39.8
Direct	0.994	0.288	0.508	1.602	60.6
Total	1.654	0.205	1.276	2.085	100.0
Weekday screen time and insomnia symptoms					
Indirect	0.820	0.194	0.427	1.193	58.1
Direct	0.590	0.226	0.187	1.090	41.9
Total	1.411	0.131	1.170	1.665	100.00
Weekend screen time and body fat percentage					
Indirect	0.590	0.178	0.244	0.969	38.0
Direct	0.964	0.244	0.505	1.485	62.0
Total	1.554	0.183	1.242	1.946	100.0
Weekend screen time and insomnia symptoms					
Indirect	0.694	0.166	0.379	1.020	51.4
Direct	0.657	0.192	0.306	1.055	48.6
Total	1.351	0.122	1.116	1.601	100.0

#### Weekend screen time and body fat percentage and insomnia symptoms

Quality of life partially mediated 38.0% of the association between weekend screen time and body fat percentage, and 51.4% of the association between weekend screen time and insomnia symptoms (Table 8).

## Discussion

The findings from this study highlighted that pre-sleep screen time, a higher quantity of screen time on a weekend, using phone as an alarm and videogaming addiction were shared determinants of a later chronotype, poor regulation of sleep onset (including irregular sleep habits, variability in sleep onset, and increased insomnia symptoms), adiposity, and obesity. These four dimensions of problematic screen time behaviour should therefore be considered when designing health-promoting interventions to improve bedtime routine, improve sleep regularity, reduce pre-sleep onset problems, and reduce adiposity.

Screen time in the 30 min before sleep onset was associated with a later chronotype and poor regulation of sleep onset (higher sleep onset variability, insomnia symptoms, poorer sleep habit), which would likely contribute to a shorter sleep duration. Evening screen time exposure has been associated with poor sleep duration across many age ranges including toddlers [71], young children [72], adolescents [72], and young adults [73]. Adolescents with excessive text-messaging whilst in bed, post-bedtime, have been shown to have a shorter sleep duration, increased daytime sleepiness and poorer academic attainment [74–77]. This is because pre-sleep screen time has been shown to suppress melatonin production due to excessive blue-light exposure [73, 78]. The exposure contributes to circadian disruption and consequently poor sleep duration and daytime sleepiness [71].

In support of the current findings, pre-sleep screen usage and excessive screen usage has been shown to be significantly associated with later chronotype [10, 35, 79], increased irregularity of sleep habits [79], increased insomnia symptoms [23, 80, 81], increased onset latency [80] and increased sleep onset variability [79]. Longitudinal research has demonstrated that an excessive quantity of screen time and screen time addiction are predictors of increased insomnia symptoms [11, 82] and adiposity [83, 84]. The effect of screen time on sleep habits, chronotype or circadian misalignment, and sleep onset variability has not been examined longitudinally to assess directionality.

Our findings showed that videogaming addiction was a shared determinant of insomnia symptoms and adiposity. The HBSC study, a large multi-country study of adolescents, found that 13–16-year-olds who were addicted to videogames had significantly later sleep onset, experienced insomnia symptoms, a longer sleep onset latency, a shorter sleep duration, and a larger social jetlag, than those not addicted to videogames [85]. Furthermore, a multi-analysis study investigating videogaming addiction across different ages within a school in the USA found that videogaming addictive tendencies can start in pre-adolescence [24]. The negative impact of videogaming addiction, however, was reported at 12 years, with adolescent’s ignoring responsibilities, reducing their sleep in order to play videogames, and neglecting physical activity and socialising outside [24].

Adolescents with videogaming addiction also engage with pre-sleep screen time, screen time in the bedroom, and excessive screen time [13, 86, 87]. Videogaming, social media, and mobile phones, have been designed to encourage digital addictiveness [86, 88, 89], and some are even designed to target adolescents. For example, social media apps target adolescents with short-form videos which have been shown to be more addictive than long-form videos and encourage ‘doom-scrolling’—the habit of compulsively scrolling through negative news or content. Consequently, trying to address the addictive tendencies of screen time is difficult. Therefore, a combined approach to an intervention, targeting multiple screen time components may be beneficial for reducing screen time addiction, and improving sleep and obesity.

The screen time variables identified as determinants of sleep and adiposity showed weekday/weekend variation, with poor weekend screen time habits being more consistently reported as shared determinants of poor sleep and adiposity than weekday habits. For example, excessive screen time on a weekend was identified as a determinant of poor sleep, which implies weekday-to-weekend variation in screen time habits could potentially contribute to unhealthy lifestyles. Previous research has shown that there are variations in screen time activity patterns on a weekday and weekend, with adolescents using excessive screen time on an evening and on a weekend [90–92]. Additionally, the variation in screen time on a weekend is more prominent in girls than boys [92], and those from a lower SES and with caregivers of a lower education level [93]. Researchers have discussed whether excessive screen time on a weekend occurs because it is being used as a parenting tool (reward system) [94], occurs due to an absence or inconsistency in the presence of parental figures (for example, opportunistic if parents work shift patterns) [94], occurs due to a lifestyle choice the adolescent makes to spend time with peers [66], and or whether it is learnt behaviour from the family [95]. Consequently, it has been suggested that interventions targeting weekend behaviour, using a family or parent-dyad setting and school-settings to target peer group social screen time behaviours.

The findings from this study indicated that adolescent wellbeing mediates the associations between different components of screen time, insomnia symptoms, and adiposity. Wellbeing accounted for over 50% of the association between weekday and weekend screen time and insomnia symptoms, compared with less than 40% for body fat percentage. A smaller mediating effect of wellbeing was observed for screen time addiction. For instance, wellbeing mediated 11% of the association between mobile phone addiction and body fat percentage, compared with 35% for insomnia symptoms. These findings suggest that pre-sleep and post-sleep screen use, as well as screen time addiction, may indirectly impair sleep onset and increase adiposity in adolescents through the impact on wellbeing.

While our findings align with previous cross-sectional studies, temporal and longitudinal research have reported mixed findings on the association between screen time and sleep outcomes [22]. Studies examining same-day screen use and subsequent sleep often find weaker associations than cross-sectional analyses [18, 96, 97]. This inconsistency may be due to differences in measurement methodologies, such as how screen time is defined (e.g. total screen time vs. specific timing of use) and the timeframe in which sleep outcomes are assessed. Temporal studies capture short-term variations in behaviour by examining same-day associations, while cross-sectional studies provide a snapshot at a single point in time, making it difficult to determine causality [98]. In contrast, longitudinal studies track behaviours over extended periods, allowing for a clearer understanding of how screen time, sleep, and adiposity interact over time [98].

Unlike same-day studies, which may underestimate the long-term effects of screen exposure, longitudinal studies provide stronger evidence that poor sleep [99–101] and obesity risks [102, 103] are influenced by multiple factors rather than screen time alone. This highlights the need for further longitudinal and experimental research to determine whether modifying screen time behaviours can improve sleep and obesity outcomes or whether screen use is a marker of underlying lifestyle and psychosocial factors.

To explore this further within the constraints of a cross-sectional study, we conducted mediation analyses to assess whether adolescent wellbeing mediates the associations between screen time, sleep, and adiposity. The results showed that once wellbeing was accounted for, the direct association between screen time and both sleep and adiposity became less pronounced, suggesting that screen time may not be the primary driver of these outcomes but instead a symptom of broader wellbeing concerns. This aligns with temporal studies indicating that the link between screen time and sleep may depend on additional factors such as psychosocial stress, lifestyle choices, and individual sleep habits.

Previous research has demonstrated that excessive screen time, particularly via smartphones and tablets, can lead to difficulties in initiating sleep due to blue light exposure and cognitive overstimulation [78, 104, 105]. This disruption in sleep onset can adversely affect adolescents' wellbeing, increasing stress and emotional distress [4, 106–108], which may in turn contribute to sedentary behaviours [109–113] poorer dietary habits [114–117], and heightened obesity risk. Given the role of wellbeing as a mediator, future interventions should not solely focus on reducing screen time but also address psychological and emotional factors that influence sleep and obesity [118, 119]. Targeted interventions could combine screen time modifications with strategies to enhance adolescent wellbeing, such as mindfulness practices, structured sleep routines, and increased physical activity [120, 121]. These approaches may provide a more comprehensive and effective strategy for mitigating sleep disturbances and obesity risks associated with screen use. Further research should evaluate the effectiveness of such multi-component interventions and explore whether addressing wellbeing alongside screen time reduction enhances adolescent health outcomes [120, 121].

Given that excessive screen use is a modifiable behaviour, promoting structured routines that reduce screen exposure in the 30 min before sleep may be a practical and scalable strategy for improving sleep duration and mitigating obesity risk in adolescents. Translating these findings into clinical practice could involve targeted public health messaging, school-based interventions, and family guidance to encourage healthier screen habits before bedtime, while also considering strategies to enhance adolescent wellbeing, as wellbeing may act as a mediator in the association between screen time and obesity.

There were multiple strengths of this study, one being the successful recruitment of participants and family members to the study, with little missing data. This indicates interest from North-east Fife for studies examining behaviours, sleep and obesity across early to late adolescence. Recruitment to the TSWS was effective, reaching the target calculated as required to perform the intended regression analyses. This was the first study to measure multiple screen time variables (timing, quantity, location, and addiction) and investigate the association with objective and subjective poor sleep and objective adiposity in adolescents. The measurement tools used for assessing the quantity of screen time and screen time addiction were validated; however, the timing and location of screen time outcomes were assessed using novel questionnaires due to these variables not being routinely assessed. A strength in our measurement of screen timing was that specific questions were asked about the first and last 30 min of the day for weekday and weekend separately, which is not often reported on in the literature. The strength of this measurement is that it gives a specific behavioural target that could be easily modified in an intervention, as opposed to minimising the quantity of screen time overall, which has been highlighted as a barrier to previous screen time interventions [8, 9]. The wide range of variables collected as part of the TSWS questionnaires and actigraphy enabled the identification of specific individual behaviours that should be considered as targets for intervention, as opposed to using latent class analysis to identifying groups of behaviours or an umbrella of behaviours (for example screen time, rather than specific measures of screen time like addiction, timing of screen time, quantity, and location of use).

A limitation of this study was that participants and caregivers, whilst recruited from the community, were from one county of Scotland (North-east Fife) and consequently, the ethnicity and SES may not be representative of the whole of Scotland. A final limitation of the TSWS was that with seven actiwatches available for the data collection, the duration of data collection (February–May) meant some participants had more favourable weather and longer daylight hours than others which could have effected their activity levels and mood [122].

A key limitation of this study is its cross-sectional design, which prevents conclusions about causality or the directionality of the associations between screen time, sleep, and adiposity. While our findings align with previous cross-sectional studies, temporal research examining same-day screen use and sleep has often reported weaker or inconsistent associations, suggesting that the impact of screen time may vary depending on additional lifestyle and psychosocial factors. Likewise, longitudinal studies tracking screen use and sleep behaviours over time indicate that multiple variables—including mental wellbeing, sedentary behaviours, and sleep hygiene—likely interact to influence adolescent sleep and adiposity outcomes.

Our findings suggest that screen exposure is one of multiple contributing factors to poor sleep and increased adiposity rather than an isolated driver. Future research should examine whether a holistic approach—modifying pre-sleep screen habits alongside strategies to enhance wellbeing, increase physical activity, and improve sleep hygiene—offers a more effective multi-component solution to improving adolescent health.

## Conclusions

The results from this study have indicated that multiple dimensions of problematic screen time behaviours, including pre-sleep and post-sleep screen time, a higher quantity of screen time (weekend), using a phone in the bedroom overnight and as an screen time addiction, should be considered as shared determinants of higher adiposity, a later chronotype and poor regulation of sleep onset. Poor quality of life mediates the association between all screen time components with insomnia symptoms and higher adiposity. Further research should consider health-promoting interventions that not only modify pre-sleep screen time usage and weekend screen time but also incorporate strategies to enhance adolescent wellbeing. Addressing factors such as stress management, mental health support, and physical activity alongside screen time reduction may provide a more effective and sustainable approach to improving adolescent sleep and reducing obesity risk. Given that excessive screen use is a modifiable behaviour, promoting structured routines that reduce screen exposure in the 30 min before sleep may be a practical and scalable strategy for improving sleep duration and mitigating obesity risk in adolescents. Translating these findings into clinical practice could involve targeted public health messaging, school-based interventions, and family guidance to encourage healthier screen habits before bedtime.

## Supplementary Information

Additional file 1: Regression. Excel spreadsheet containing full regression analysis outputs, including coefficients, standard errors, confidence intervals, and significance values for each screen time variable in relation to sleep and adiposity outcomes in the TSWS dataset.

Additional file 2: Mediation code. R script containing all code used to conduct the mediation analysis component of this paper.

## Data Availability

The original raw datasets analysed during the current study are not publicly available due to data sharing conditions outlined in the participant consent. Although fully anonymised, ethical approval did not permit public data storing and sharing. Requests to access the datasets should be directed to the corresponding author. Additional file 1 contains the full regression analyses, and Additional file 2 contains the R code used for mediation analyses.

## Abbreviations

AdjR2: Adjusted coefficient of determination
BF%: Body fat percentage
BMIp: Body Mass Index percentile
CI: Confidence interval
CSHQ: Children’s Sleep Habits Questionnaire
DV: Dependent variable
ISI: Insomnia Severity Index
MCAR: Missing Completely At Random
MEQ: Morningness–Eveningness Questionnaire
MPA-Q: Mobile Phone Addiction Questionnaire
OF/OB: Over-fat/obese
QoL: Quality of life
*R*^2^: Coefficient of determination
SCREENS-Q: Screen Media Use in Children Questionnaire
SD: Standard deviation
SE: Standard error
SIMD: Scottish Index of Multiple Deprivation
SMA-Q: Social Media Addiction Questionnaire
TSWS: Teen Sleep Well Study
UF/HF: Under-fat/healthy-fat
VGA-Q: Videogaming Addiction Questionnaire
WD: Weekday
WE: Weekend
