# Supplementary material for: Pre-sleep screen time and screen time addiction as shared determinants of poor sleep and obesity in adolescents aged 11–14 years in Scotland
Source: BMC Glob Public Health. 2025 May 7;3:42. doi: 10.1186/s44263-025-00160-y (PMC12060382; doi:10.1186/s44263-025-00160-y)
Supplement: Supplementary file 2 — Additional file 2: Mediation code. R script containing all code used to conduct the mediation analysis component of this paper. [file 44263_2025_160_MOESM2_ESM.docx]

**Additional file 2 – Mediation code**

Description: R script containing all code used to conduct the mediation analysis component of this paper.

**# Mediation of QOL on the relationship between BF and Late night ST**

# Load necessary libraries

library(lavaan)

library(dplyr)

# Load your dataset

TSWS_master_data_set <- read.csv("path_to_your_file/TSWS_master_data_set.csv")

# Define the mediation model in lavaan syntax

model <- '

# Mediator model

MWqol ~ a*LNtiming

# Outcome model

ADbodyfat ~ b*MWqol + c*LNtiming

# Indirect effect

indirect := a*b

# Direct effect

direct := c

# Total effect

total := c + (a*b)

'

# Fit the model using the lavaan function

fit <- sem(model, data = TSWS_master_data_set, se = "bootstrap", bootstrap = 1000)

# Print a summary of the results

summary(fit, standardized = TRUE, fit.measures = TRUE, rsquare = TRUE)

# Extract path coefficients and standard errors

results <- parameterEstimates(fit, standardized = TRUE, ci = TRUE)

results <- results[results$op %in% c("~", ":="), ] # Filter relevant results

# Print the results to check contents and available labels

print(results)

print(unique(results$label))

# Initialize empty vectors for the table

effect_labels <- c("Controlled Direct Effect (CDE)", "Natural Indirect Effect (NIE)", "Total Effect")

estimates <- numeric(length(effect_labels))

standard_errors <- numeric(length(effect_labels))

lower_ci <- numeric(length(effect_labels))

upper_ci <- numeric(length(effect_labels))

p_values <- numeric(length(effect_labels))

# Extract relevant values for each effect

for (i in seq_along(effect_labels)) {

label <- switch(effect_labels[i],

"Controlled Direct Effect (CDE)" = "direct",

"Natural Indirect Effect (NIE)" = "indirect",

"Total Effect" = "total"

)

# Check if the label exists in results

if (label %in% results$label) {

effect_row <- results[results$label == label, ]

if (nrow(effect_row) > 0) {

estimates[i] <- effect_row$est.std[1] # Take the first element if there are multiple

standard_errors[i] <- effect_row$se[1]

lower_ci[i] <- effect_row$ci.lower[1]

upper_ci[i] <- effect_row$ci.upper[1]

p_values[i] <- effect_row$pvalue[1]

} else {

estimates[i] <- NA

standard_errors[i] <- NA

lower_ci[i] <- NA

upper_ci[i] <- NA

p_values[i] <- NA

}

} else {

estimates[i] <- NA

standard_errors[i] <- NA

lower_ci[i] <- NA

upper_ci[i] <- NA

p_values[i] <- NA

}

}

# Create a table to summarize the effects

mediation_table <- data.frame(

Effect = effect_labels,

Estimate = estimates,

Standard_Error = standard_errors,

Lower_CI = lower_ci,

Upper_CI = upper_ci,

p_value = p_values

)

# Display the table

print(mediation_table)

**#Mediation of QOL on the relationship between Insomnia symptoms and Late night ST**

# Load necessary libraries

library(lavaan)

library(dplyr)

# Load your dataset

TSWS_master_data_set <- read.csv("path_to_your_file/TSWS_master_data_set.csv")

# Define the mediation model in lavaan syntax

model <- '

# Mediator model

MWqol ~ a*LNtiming

# Outcome model

PREisiscore ~ b*MWqol + c*LNtiming

# Indirect effect

indirect := a*b

# Direct effect

direct := c

# Total effect

total := c + (a*b)

'

# Fit the model using the lavaan function

fit <- sem(model, data = TSWS_master_data_set, se = "bootstrap", bootstrap = 1000)

# Print a summary of the results

summary(fit, standardized = TRUE, fit.measures = TRUE, rsquare = TRUE)

# Extract path coefficients and standard errors

results <- parameterEstimates(fit, standardized = TRUE, ci = TRUE)

results <- results[results$op %in% c("~", ":="), ] # Filter relevant results

# Print the results to check contents and available labels

print(results)

print(unique(results$label))

# Initialize empty vectors for the table

effect_labels <- c("Controlled Direct Effect (CDE)", "Natural Indirect Effect (NIE)", "Total Effect")

estimates <- numeric(length(effect_labels))

standard_errors <- numeric(length(effect_labels))

lower_ci <- numeric(length(effect_labels))

upper_ci <- numeric(length(effect_labels))

p_values <- numeric(length(effect_labels))

# Extract relevant values for each effect

for (i in seq_along(effect_labels)) {

label <- switch(effect_labels[i],

"Controlled Direct Effect (CDE)" = "direct",

"Natural Indirect Effect (NIE)" = "indirect",

"Total Effect" = "total"

)

# Check if the label exists in results

if (label %in% results$label) {

effect_row <- results[results$label == label, ]

if (nrow(effect_row) > 0) {

estimates[i] <- effect_row$est.std[1] # Take the first element if there are multiple

standard_errors[i] <- effect_row$se[1]

lower_ci[i] <- effect_row$ci.lower[1]

upper_ci[i] <- effect_row$ci.upper[1]

p_values[i] <- effect_row$pvalue[1]

} else {

estimates[i] <- NA

standard_errors[i] <- NA

lower_ci[i] <- NA

upper_ci[i] <- NA

p_values[i] <- NA

}

} else {

estimates[i] <- NA

standard_errors[i] <- NA

lower_ci[i] <- NA

upper_ci[i] <- NA

p_values[i] <- NA

}

}

# Create a table to summarize the effects

mediation_table <- data.frame(

Effect = effect_labels,

Estimate = estimates,

Standard_Error = standard_errors,

Lower_CI = lower_ci,

Upper_CI = upper_ci,

p_value = p_values

)

# Display the table

print(mediation_table)

**# Mediation of QOL on the relationship between BF and Early morning ST**

# Load necessary libraries

library(lavaan)

library(dplyr)

# Load your dataset

TSWS_master_data_set <- read.csv("path_to_your_file/TSWS_master_data_set.csv")

# Define the mediation model in lavaan syntax

model <- '

# Mediator model

MWqol ~ a*EMtiming

# Outcome model

ADbodyfat ~ b*MWqol + c*EMtiming

# Indirect effect

indirect := a*b

# Direct effect

direct := c

# Total effect

total := c + (a*b)

'

# Fit the model using the lavaan function

fit <- sem(model, data = TSWS_master_data_set, se = "bootstrap", bootstrap = 1000)

# Print a summary of the results

summary(fit, standardized = TRUE, fit.measures = TRUE, rsquare = TRUE)

# Extract path coefficients and standard errors

results <- parameterEstimates(fit, standardized = TRUE, ci = TRUE)

results <- results[results$op %in% c("~", ":="), ] # Filter relevant results

# Print the results to check contents and available labels

print(results)

print(unique(results$label))

# Initialize empty vectors for the table

effect_labels <- c("Controlled Direct Effect (CDE)", "Natural Indirect Effect (NIE)", "Total Effect")

estimates <- numeric(length(effect_labels))

standard_errors <- numeric(length(effect_labels))

lower_ci <- numeric(length(effect_labels))

upper_ci <- numeric(length(effect_labels))

p_values <- numeric(length(effect_labels))

# Extract relevant values for each effect

for (i in seq_along(effect_labels)) {

label <- switch(effect_labels[i],

"Controlled Direct Effect (CDE)" = "direct",

"Natural Indirect Effect (NIE)" = "indirect",

"Total Effect" = "total"

)

# Check if the label exists in results

if (label %in% results$label) {

effect_row <- results[results$label == label, ]

if (nrow(effect_row) > 0) {

estimates[i] <- effect_row$est.std[1] # Take the first element if there are multiple

standard_errors[i] <- effect_row$se[1]

lower_ci[i] <- effect_row$ci.lower[1]

upper_ci[i] <- effect_row$ci.upper[1]

p_values[i] <- effect_row$pvalue[1]

} else {

estimates[i] <- NA

standard_errors[i] <- NA

lower_ci[i] <- NA

upper_ci[i] <- NA

p_values[i] <- NA

}

} else {

estimates[i] <- NA

standard_errors[i] <- NA

lower_ci[i] <- NA

upper_ci[i] <- NA

p_values[i] <- NA

}

}

# Create a table to summarize the effects

mediation_table <- data.frame(

Effect = effect_labels,

Estimate = estimates,

Standard_Error = standard_errors,

Lower_CI = lower_ci,

Upper_CI = upper_ci,

p_value = p_values

)

# Display the table

print(mediation_table)

**#Mediation of QOL on the relationship between Insomnia symptoms and Late night ST**

# Load necessary libraries

library(lavaan)

library(dplyr)

# Load your dataset

TSWS_master_data_set <- read.csv("path_to_your_file/TSWS_master_data_set.csv")

# Define the mediation model in lavaan syntax

model <- '

# Mediator model

MWqol ~ a*EMtiming

# Outcome model

PREisiscore ~ b*MWqol + c*EMtiming

# Indirect effect

indirect := a*b

# Direct effect

direct := c

# Total effect

total := c + (a*b)

'

# Fit the model using the lavaan function

fit <- sem(model, data = TSWS_master_data_set, se = "bootstrap", bootstrap = 1000)

# Print a summary of the results

summary(fit, standardized = TRUE, fit.measures = TRUE, rsquare = TRUE)

# Extract path coefficients and standard errors

results <- parameterEstimates(fit, standardized = TRUE, ci = TRUE)

results <- results[results$op %in% c("~", ":="), ] # Filter relevant results

# Print the results to check contents and available labels

print(results)

print(unique(results$label))

# Initialize empty vectors for the table

effect_labels <- c("Controlled Direct Effect (CDE)", "Natural Indirect Effect (NIE)", "Total Effect")

estimates <- numeric(length(effect_labels))

standard_errors <- numeric(length(effect_labels))

lower_ci <- numeric(length(effect_labels))

upper_ci <- numeric(length(effect_labels))

p_values <- numeric(length(effect_labels))

# Extract relevant values for each effect

for (i in seq_along(effect_labels)) {

label <- switch(effect_labels[i],

"Controlled Direct Effect (CDE)" = "direct",

"Natural Indirect Effect (NIE)" = "indirect",

"Total Effect" = "total"

)

# Check if the label exists in results

if (label %in% results$label) {

effect_row <- results[results$label == label, ]

if (nrow(effect_row) > 0) {

estimates[i] <- effect_row$est.std[1] # Take the first element if there are multiple

standard_errors[i] <- effect_row$se[1]

lower_ci[i] <- effect_row$ci.lower[1]

upper_ci[i] <- effect_row$ci.upper[1]

p_values[i] <- effect_row$pvalue[1]

} else {

estimates[i] <- NA

standard_errors[i] <- NA

lower_ci[i] <- NA

upper_ci[i] <- NA

p_values[i] <- NA

}

} else {

estimates[i] <- NA

standard_errors[i] <- NA

lower_ci[i] <- NA

upper_ci[i] <- NA

p_values[i] <- NA

}

}

# Create a table to summarize the effects

mediation_table <- data.frame(

Effect = effect_labels,

Estimate = estimates,

Standard_Error = standard_errors,

Lower_CI = lower_ci,

Upper_CI = upper_ci,

p_value = p_values

)

# Display the table

print(mediation_table)

**# Mediation of QOL on the relationship between Insomnia symptoms and Videogaming**

# Load necessary libraries

library(lavaan)

library(dplyr)

# Load your dataset

TSWS_master_data_set <- read.csv("path_to_your_file/TSWS_master_data_set.csv")

# Define the mediation model in lavaan syntax

model <- '

# Mediator model

MWqol ~ a*STvideogaming

# Outcome model

PREisiscore ~ b*MWqol + c*STvideogaming

# Indirect effect

indirect := a*b

# Direct effect

direct := c

# Total effect

total := c + (a*b)

'

# Fit the model using the lavaan function

fit <- sem(model, data = TSWS_master_data_set, se = "bootstrap", bootstrap = 1000)

# Print a summary of the results

summary(fit, standardized = TRUE, fit.measures = TRUE, rsquare = TRUE)

# Extract path coefficients and standard errors

results <- parameterEstimates(fit, standardized = TRUE, ci = TRUE)

results <- results[results$op %in% c("~", ":="), ] # Filter relevant results

# Print the results to check contents and available labels

print(results)

print(unique(results$label))

# Initialize empty vectors for the table

effect_labels <- c("Controlled Direct Effect (CDE)", "Natural Indirect Effect (NIE)", "Total Effect")

estimates <- numeric(length(effect_labels))

standard_errors <- numeric(length(effect_labels))

lower_ci <- numeric(length(effect_labels))

upper_ci <- numeric(length(effect_labels))

p_values <- numeric(length(effect_labels))

# Extract relevant values for each effect

for (i in seq_along(effect_labels)) {

label <- switch(effect_labels[i],

"Controlled Direct Effect (CDE)" = "direct",

"Natural Indirect Effect (NIE)" = "indirect",

"Total Effect" = "total"

)

# Check if the label exists in results

if (label %in% results$label) {

effect_row <- results[results$label == label, ]

if (nrow(effect_row) > 0) {

estimates[i] <- effect_row$est.std[1] # Take the first element if there are multiple

standard_errors[i] <- effect_row$se[1]

lower_ci[i] <- effect_row$ci.lower[1]

upper_ci[i] <- effect_row$ci.upper[1]

p_values[i] <- effect_row$pvalue[1]

} else {

estimates[i] <- NA

standard_errors[i] <- NA

lower_ci[i] <- NA

upper_ci[i] <- NA

p_values[i] <- NA

}

} else {

estimates[i] <- NA

standard_errors[i] <- NA

lower_ci[i] <- NA

upper_ci[i] <- NA

p_values[i] <- NA

}

}

# Create a table to summarize the effects

mediation_table <- data.frame(

Effect = effect_labels,

Estimate = estimates,

Standard_Error = standard_errors,

Lower_CI = lower_ci,

Upper_CI = upper_ci,

p_value = p_values

)

# Display the table

print(mediation_table)

**# Mediation of QOL on the relationship between BF and Videogaming**

# Load necessary libraries

library(lavaan)

library(dplyr)

# Load your dataset

TSWS_master_data_set <- read.csv("path_to_your_file/TSWS_master_data_set.csv")

# Define the mediation model in lavaan syntax

model <- '

# Mediator model

MWqol ~ a*STvideogaming

# Outcome model

ADbodyfat ~ b*MWqol + c*STvideogaming

# Indirect effect

indirect := a*b

# Direct effect

direct := c

# Total effect

total := c + (a*b)

'

# Fit the model using the lavaan function

fit <- sem(model, data = TSWS_master_data_set, se = "bootstrap", bootstrap = 1000)

# Print a summary of the results

summary(fit, standardized = TRUE, fit.measures = TRUE, rsquare = TRUE)

# Extract path coefficients and standard errors

results <- parameterEstimates(fit, standardized = TRUE, ci = TRUE)

results <- results[results$op %in% c("~", ":="), ] # Filter relevant results

# Print the results to check contents and available labels

print(results)

print(unique(results$label))

# Initialize empty vectors for the table

effect_labels <- c("Controlled Direct Effect (CDE)", "Natural Indirect Effect (NIE)", "Total Effect")

estimates <- numeric(length(effect_labels))

standard_errors <- numeric(length(effect_labels))

lower_ci <- numeric(length(effect_labels))

upper_ci <- numeric(length(effect_labels))

p_values <- numeric(length(effect_labels))

# Extract relevant values for each effect

for (i in seq_along(effect_labels)) {

label <- switch(effect_labels[i],

"Controlled Direct Effect (CDE)" = "direct",

"Natural Indirect Effect (NIE)" = "indirect",

"Total Effect" = "total"

)

# Check if the label exists in results

if (label %in% results$label) {

effect_row <- results[results$label == label, ]

if (nrow(effect_row) > 0) {

estimates[i] <- effect_row$est.std[1] # Take the first element if there are multiple

standard_errors[i] <- effect_row$se[1]

lower_ci[i] <- effect_row$ci.lower[1]

upper_ci[i] <- effect_row$ci.upper[1]

p_values[i] <- effect_row$pvalue[1]

} else {

estimates[i] <- NA

standard_errors[i] <- NA

lower_ci[i] <- NA

upper_ci[i] <- NA

p_values[i] <- NA

}

} else {

estimates[i] <- NA

standard_errors[i] <- NA

lower_ci[i] <- NA

upper_ci[i] <- NA

p_values[i] <- NA

}

}

# Create a table to summarize the effects

mediation_table <- data.frame(

Effect = effect_labels,

Estimate = estimates,

Standard_Error = standard_errors,

Lower_CI = lower_ci,

Upper_CI = upper_ci,

p_value = p_values

)

# Display the table

print(mediation_table)

**# Mediation of QOL on the relationship between BF and Social media**

# Load necessary libraries

library(lavaan)

library(dplyr)

# Load your dataset

TSWS_master_data_set <- read.csv("path_to_your_file/TSWS_master_data_set.csv")

# Define the mediation model in lavaan syntax

model <- '

# Mediator model

MWqol ~ a*STsocialmedia

# Outcome model

ADbodyfat ~ b*MWqol + c*STsocialmedia

# Indirect effect

indirect := a*b

# Direct effect

direct := c

# Total effect

total := c + (a*b)

'

# Fit the model using the lavaan function

fit <- sem(model, data = TSWS_master_data_set, se = "bootstrap", bootstrap = 1000)

# Print a summary of the results

summary(fit, standardized = TRUE, fit.measures = TRUE, rsquare = TRUE)

# Extract path coefficients and standard errors

results <- parameterEstimates(fit, standardized = TRUE, ci = TRUE)

results <- results[results$op %in% c("~", ":="), ] # Filter relevant results

# Print the results to check contents and available labels

print(results)

print(unique(results$label))

# Initialize empty vectors for the table

effect_labels <- c("Controlled Direct Effect (CDE)", "Natural Indirect Effect (NIE)", "Total Effect")

estimates <- numeric(length(effect_labels))

standard_errors <- numeric(length(effect_labels))

lower_ci <- numeric(length(effect_labels))

upper_ci <- numeric(length(effect_labels))

p_values <- numeric(length(effect_labels))

# Extract relevant values for each effect

for (i in seq_along(effect_labels)) {

label <- switch(effect_labels[i],

"Controlled Direct Effect (CDE)" = "direct",

"Natural Indirect Effect (NIE)" = "indirect",

"Total Effect" = "total"

)

# Check if the label exists in results

if (label %in% results$label) {

effect_row <- results[results$label == label, ]

if (nrow(effect_row) > 0) {

estimates[i] <- effect_row$est.std[1] # Take the first element if there are multiple

standard_errors[i] <- effect_row$se[1]

lower_ci[i] <- effect_row$ci.lower[1]

upper_ci[i] <- effect_row$ci.upper[1]

p_values[i] <- effect_row$pvalue[1]

} else {

estimates[i] <- NA

standard_errors[i] <- NA

lower_ci[i] <- NA

upper_ci[i] <- NA

p_values[i] <- NA

}

} else {

estimates[i] <- NA

standard_errors[i] <- NA

lower_ci[i] <- NA

upper_ci[i] <- NA

p_values[i] <- NA

}

}

# Create a table to summarize the effects

mediation_table <- data.frame(

Effect = effect_labels,

Estimate = estimates,

Standard_Error = standard_errors,

Lower_CI = lower_ci,

Upper_CI = upper_ci,

p_value = p_values

)

# Display the table

print(mediation_table)

**# Mediation of QOL on the relationship between insomnia symptoms and Social media**

# Load necessary libraries

library(lavaan)

library(dplyr)

# Load your dataset

TSWS_master_data_set <- read.csv("path_to_your_file/TSWS_master_data_set.csv")

# Define the mediation model in lavaan syntax

model <- '

# Mediator model

MWqol ~ a*STsocialmedia

# Outcome model

PREisiscore ~ b*MWqol + c*STsocialmedia

# Indirect effect

indirect := a*b

# Direct effect

direct := c

# Total effect

total := c + (a*b)

'

# Fit the model using the lavaan function

fit <- sem(model, data = TSWS_master_data_set, se = "bootstrap", bootstrap = 1000)

# Print a summary of the results

summary(fit, standardized = TRUE, fit.measures = TRUE, rsquare = TRUE)

# Extract path coefficients and standard errors

results <- parameterEstimates(fit, standardized = TRUE, ci = TRUE)

results <- results[results$op %in% c("~", ":="), ] # Filter relevant results

# Print the results to check contents and available labels

print(results)

print(unique(results$label))

# Initialize empty vectors for the table

effect_labels <- c("Controlled Direct Effect (CDE)", "Natural Indirect Effect (NIE)", "Total Effect")

estimates <- numeric(length(effect_labels))

standard_errors <- numeric(length(effect_labels))

lower_ci <- numeric(length(effect_labels))

upper_ci <- numeric(length(effect_labels))

p_values <- numeric(length(effect_labels))

# Extract relevant values for each effect

for (i in seq_along(effect_labels)) {

label <- switch(effect_labels[i],

"Controlled Direct Effect (CDE)" = "direct",

"Natural Indirect Effect (NIE)" = "indirect",

"Total Effect" = "total"

)

# Check if the label exists in results

if (label %in% results$label) {

effect_row <- results[results$label == label, ]

if (nrow(effect_row) > 0) {

estimates[i] <- effect_row$est.std[1] # Take the first element if there are multiple

standard_errors[i] <- effect_row$se[1]

lower_ci[i] <- effect_row$ci.lower[1]

upper_ci[i] <- effect_row$ci.upper[1]

p_values[i] <- effect_row$pvalue[1]

} else {

estimates[i] <- NA

standard_errors[i] <- NA

lower_ci[i] <- NA

upper_ci[i] <- NA

p_values[i] <- NA

}

} else {

estimates[i] <- NA

standard_errors[i] <- NA

lower_ci[i] <- NA

upper_ci[i] <- NA

p_values[i] <- NA

}

}

# Create a table to summarize the effects

mediation_table <- data.frame(

Effect = effect_labels,

Estimate = estimates,

Standard_Error = standard_errors,

Lower_CI = lower_ci,

Upper_CI = upper_ci,

p_value = p_values

)

# Display the table

print(mediation_table)

**# Mediation of QOL on the relationship between insomnia symptoms and mobile phone**

# Load necessary libraries

library(lavaan)

library(dplyr)

# Load your dataset

TSWS_master_data_set <- read.csv("path_to_your_file/TSWS_master_data_set.csv")

# Define the mediation model in lavaan syntax

model <- '

# Mediator model

MWqol ~ a*STmobilephone

# Outcome model

PREisiscore ~ b*MWqol + c*STmobilephone

# Indirect effect

indirect := a*b

# Direct effect

direct := c

# Total effect

total := c + (a*b)

'

# Fit the model using the lavaan function

fit <- sem(model, data = TSWS_master_data_set, se = "bootstrap", bootstrap = 1000)

# Print a summary of the results

summary(fit, standardized = TRUE, fit.measures = TRUE, rsquare = TRUE)

# Extract path coefficients and standard errors

results <- parameterEstimates(fit, standardized = TRUE, ci = TRUE)

results <- results[results$op %in% c("~", ":="), ] # Filter relevant results

# Print the results to check contents and available labels

print(results)

print(unique(results$label))

# Initialize empty vectors for the table

effect_labels <- c("Controlled Direct Effect (CDE)", "Natural Indirect Effect (NIE)", "Total Effect")

estimates <- numeric(length(effect_labels))

standard_errors <- numeric(length(effect_labels))

lower_ci <- numeric(length(effect_labels))

upper_ci <- numeric(length(effect_labels))

p_values <- numeric(length(effect_labels))

# Extract relevant values for each effect

for (i in seq_along(effect_labels)) {

label <- switch(effect_labels[i],

"Controlled Direct Effect (CDE)" = "direct",

"Natural Indirect Effect (NIE)" = "indirect",

"Total Effect" = "total"

)

# Check if the label exists in results

if (label %in% results$label) {

effect_row <- results[results$label == label, ]

if (nrow(effect_row) > 0) {

estimates[i] <- effect_row$est.std[1] # Take the first element if there are multiple

standard_errors[i] <- effect_row$se[1]

lower_ci[i] <- effect_row$ci.lower[1]

upper_ci[i] <- effect_row$ci.upper[1]

p_values[i] <- effect_row$pvalue[1]

} else {

estimates[i] <- NA

standard_errors[i] <- NA

lower_ci[i] <- NA

upper_ci[i] <- NA

p_values[i] <- NA

}

} else {

estimates[i] <- NA

standard_errors[i] <- NA

lower_ci[i] <- NA

upper_ci[i] <- NA

p_values[i] <- NA

}

}

# Create a table to summarize the effects

mediation_table <- data.frame(

Effect = effect_labels,

Estimate = estimates,

Standard_Error = standard_errors,

Lower_CI = lower_ci,

Upper_CI = upper_ci,

p_value = p_values

)

# Display the table

print(mediation_table)

**# Mediation of QOL on the relationship between BF and mobile phone**

# Load necessary libraries

library(lavaan)

library(dplyr)

# Load your dataset

TSWS_master_data_set <- read.csv("path_to_your_file/TSWS_master_data_set.csv")

# Define the mediation model in lavaan syntax

model <- '

# Mediator model

MWqol ~ a*STmobilephone

# Outcome model

ADbodyfat ~ b*MWqol + c*STmobilephone

# Indirect effect

indirect := a*b

# Direct effect

direct := c

# Total effect

total := c + (a*b)

'

# Fit the model using the lavaan function

fit <- sem(model, data = TSWS_master_data_set, se = "bootstrap", bootstrap = 1000)

# Print a summary of the results

summary(fit, standardized = TRUE, fit.measures = TRUE, rsquare = TRUE)

# Extract path coefficients and standard errors

results <- parameterEstimates(fit, standardized = TRUE, ci = TRUE)

results <- results[results$op %in% c("~", ":="), ] # Filter relevant results

# Print the results to check contents and available labels

print(results)

print(unique(results$label))

# Initialize empty vectors for the table

effect_labels <- c("Controlled Direct Effect (CDE)", "Natural Indirect Effect (NIE)", "Total Effect")

estimates <- numeric(length(effect_labels))

standard_errors <- numeric(length(effect_labels))

lower_ci <- numeric(length(effect_labels))

upper_ci <- numeric(length(effect_labels))

p_values <- numeric(length(effect_labels))

# Extract relevant values for each effect

for (i in seq_along(effect_labels)) {

label <- switch(effect_labels[i],

"Controlled Direct Effect (CDE)" = "direct",

"Natural Indirect Effect (NIE)" = "indirect",

"Total Effect" = "total"

)

# Check if the label exists in results

if (label %in% results$label) {

effect_row <- results[results$label == label, ]

if (nrow(effect_row) > 0) {

estimates[i] <- effect_row$est.std[1] # Take the first element if there are multiple

standard_errors[i] <- effect_row$se[1]

lower_ci[i] <- effect_row$ci.lower[1]

upper_ci[i] <- effect_row$ci.upper[1]

p_values[i] <- effect_row$pvalue[1]

} else {

estimates[i] <- NA

standard_errors[i] <- NA

lower_ci[i] <- NA

upper_ci[i] <- NA

p_values[i] <- NA

}

} else {

estimates[i] <- NA

standard_errors[i] <- NA

lower_ci[i] <- NA

upper_ci[i] <- NA

p_values[i] <- NA

}

}

# Create a table to summarize the effects

mediation_table <- data.frame(

Effect = effect_labels,

Estimate = estimates,

Standard_Error = standard_errors,

Lower_CI = lower_ci,

Upper_CI = upper_ci,

p_value = p_values

)

# Display the table

print(mediation_table)

**# Mediation of QOL on the relationship between BF and ST quantity WD**

# Load necessary libraries

library(lavaan)

library(dplyr)

# Load your dataset

TSWS_master_data_set <- read.csv("path_to_your_file/TSWS_master_data_set.csv")

# Define the mediation model in lavaan syntax

model <- '

# Mediator model

MWqol ~ a*STquantitywd

# Outcome model

ADbodyfat ~ b*MWqol + c*STquantitywd

# Indirect effect

indirect := a*b

# Direct effect

direct := c

# Total effect

total := c + (a*b)

'

# Fit the model using the lavaan function

fit <- sem(model, data = TSWS_master_data_set, se = "bootstrap", bootstrap = 1000)

# Print a summary of the results

summary(fit, standardized = TRUE, fit.measures = TRUE, rsquare = TRUE)

# Extract path coefficients and standard errors

results <- parameterEstimates(fit, standardized = TRUE, ci = TRUE)

results <- results[results$op %in% c("~", ":="), ] # Filter relevant results

# Print the results to check contents and available labels

print(results)

print(unique(results$label))

# Initialize empty vectors for the table

effect_labels <- c("Controlled Direct Effect (CDE)", "Natural Indirect Effect (NIE)", "Total Effect")

estimates <- numeric(length(effect_labels))

standard_errors <- numeric(length(effect_labels))

lower_ci <- numeric(length(effect_labels))

upper_ci <- numeric(length(effect_labels))

p_values <- numeric(length(effect_labels))

# Extract relevant values for each effect

for (i in seq_along(effect_labels)) {

label <- switch(effect_labels[i],

"Controlled Direct Effect (CDE)" = "direct",

"Natural Indirect Effect (NIE)" = "indirect",

"Total Effect" = "total"

)

# Check if the label exists in results

if (label %in% results$label) {

effect_row <- results[results$label == label, ]

if (nrow(effect_row) > 0) {

estimates[i] <- effect_row$est.std[1] # Take the first element if there are multiple

standard_errors[i] <- effect_row$se[1]

lower_ci[i] <- effect_row$ci.lower[1]

upper_ci[i] <- effect_row$ci.upper[1]

p_values[i] <- effect_row$pvalue[1]

} else {

estimates[i] <- NA

standard_errors[i] <- NA

lower_ci[i] <- NA

upper_ci[i] <- NA

p_values[i] <- NA

}

} else {

estimates[i] <- NA

standard_errors[i] <- NA

lower_ci[i] <- NA

upper_ci[i] <- NA

p_values[i] <- NA

}

}

# Create a table to summarize the effects

mediation_table <- data.frame(

Effect = effect_labels,

Estimate = estimates,

Standard_Error = standard_errors,

Lower_CI = lower_ci,

Upper_CI = upper_ci,

p_value = p_values

)

# Display the table

print(mediation_table)

**# Mediation of QOL on the relationship between insomnia symptoms and ST quantity WD**

# Load necessary libraries

library(lavaan)

library(dplyr)

# Load your dataset

TSWS_master_data_set <- read.csv("path_to_your_file/TSWS_master_data_set.csv")

# Define the mediation model in lavaan syntax

model <- '

# Mediator model

MWqol ~ a*STquantitywd

# Outcome model

PREisiscore ~ b*MWqol + c*STquantitywd

# Indirect effect

indirect := a*b

# Direct effect

direct := c

# Total effect

total := c + (a*b)

'

# Fit the model using the lavaan function

fit <- sem(model, data = TSWS_master_data_set, se = "bootstrap", bootstrap = 1000)

# Print a summary of the results

summary(fit, standardized = TRUE, fit.measures = TRUE, rsquare = TRUE)

# Extract path coefficients and standard errors

results <- parameterEstimates(fit, standardized = TRUE, ci = TRUE)

results <- results[results$op %in% c("~", ":="), ] # Filter relevant results

# Print the results to check contents and available labels

print(results)

print(unique(results$label))

# Initialize empty vectors for the table

effect_labels <- c("Controlled Direct Effect (CDE)", "Natural Indirect Effect (NIE)", "Total Effect")

estimates <- numeric(length(effect_labels))

standard_errors <- numeric(length(effect_labels))

lower_ci <- numeric(length(effect_labels))

upper_ci <- numeric(length(effect_labels))

p_values <- numeric(length(effect_labels))

# Extract relevant values for each effect

for (i in seq_along(effect_labels)) {

label <- switch(effect_labels[i],

"Controlled Direct Effect (CDE)" = "direct",

"Natural Indirect Effect (NIE)" = "indirect",

"Total Effect" = "total"

)

# Check if the label exists in results

if (label %in% results$label) {

effect_row <- results[results$label == label, ]

if (nrow(effect_row) > 0) {

estimates[i] <- effect_row$est.std[1] # Take the first element if there are multiple

standard_errors[i] <- effect_row$se[1]

lower_ci[i] <- effect_row$ci.lower[1]

upper_ci[i] <- effect_row$ci.upper[1]

p_values[i] <- effect_row$pvalue[1]

} else {

estimates[i] <- NA

standard_errors[i] <- NA

lower_ci[i] <- NA

upper_ci[i] <- NA

p_values[i] <- NA

}

} else {

estimates[i] <- NA

standard_errors[i] <- NA

lower_ci[i] <- NA

upper_ci[i] <- NA

p_values[i] <- NA

}

}

# Create a table to summarize the effects

mediation_table <- data.frame(

Effect = effect_labels,

Estimate = estimates,

Standard_Error = standard_errors,

Lower_CI = lower_ci,

Upper_CI = upper_ci,

p_value = p_values

)

# Display the table

print(mediation_table)

**# Mediation of QOL on the relationship between insomnia symptoms and ST quantity WE**

# Load necessary libraries

library(lavaan)

library(dplyr)

# Load your dataset

TSWS_master_data_set <- read.csv("path_to_your_file/TSWS_master_data_set.csv")

# Define the mediation model in lavaan syntax

model <- '

# Mediator model

MWqol ~ a*STquantitywe

# Outcome model

PREisiscore ~ b*MWqol + c*STquantitywe

# Indirect effect

indirect := a*b

# Direct effect

direct := c

# Total effect

total := c + (a*b)

'

# Fit the model using the lavaan function

fit <- sem(model, data = TSWS_master_data_set, se = "bootstrap", bootstrap = 1000)

# Print a summary of the results

summary(fit, standardized = TRUE, fit.measures = TRUE, rsquare = TRUE)

# Extract path coefficients and standard errors

results <- parameterEstimates(fit, standardized = TRUE, ci = TRUE)

results <- results[results$op %in% c("~", ":="), ] # Filter relevant results

# Print the results to check contents and available labels

print(results)

print(unique(results$label))

# Initialize empty vectors for the table

effect_labels <- c("Controlled Direct Effect (CDE)", "Natural Indirect Effect (NIE)", "Total Effect")

estimates <- numeric(length(effect_labels))

standard_errors <- numeric(length(effect_labels))

lower_ci <- numeric(length(effect_labels))

upper_ci <- numeric(length(effect_labels))

p_values <- numeric(length(effect_labels))

# Extract relevant values for each effect

for (i in seq_along(effect_labels)) {

label <- switch(effect_labels[i],

"Controlled Direct Effect (CDE)" = "direct",

"Natural Indirect Effect (NIE)" = "indirect",

"Total Effect" = "total"

)

# Check if the label exists in results

if (label %in% results$label) {

effect_row <- results[results$label == label, ]

if (nrow(effect_row) > 0) {

estimates[i] <- effect_row$est.std[1] # Take the first element if there are multiple

standard_errors[i] <- effect_row$se[1]

lower_ci[i] <- effect_row$ci.lower[1]

upper_ci[i] <- effect_row$ci.upper[1]

p_values[i] <- effect_row$pvalue[1]

} else {

estimates[i] <- NA

standard_errors[i] <- NA

lower_ci[i] <- NA

upper_ci[i] <- NA

p_values[i] <- NA

}

} else {

estimates[i] <- NA

standard_errors[i] <- NA

lower_ci[i] <- NA

**# Mediation of QOL on the relationship between BF and ST quantity WE**

# Load necessary libraries

library(lavaan)

library(dplyr)

# Load your dataset

TSWS_master_data_set <- read.csv("path_to_your_file/TSWS_master_data_set.csv")

# Define the mediation model in lavaan syntax

model <- '

# Mediator model

MWqol ~ a*STquantitywe

# Outcome model

ADbodyfat ~ b*MWqol + c*STquantitywe

# Indirect effect

indirect := a*b

# Direct effect

direct := c

# Total effect

total := c + (a*b)

'

# Fit the model using the lavaan function

fit <- sem(model, data = TSWS_master_data_set, se = "bootstrap", bootstrap = 1000)

# Print a summary of the results

summary(fit, standardized = TRUE, fit.measures = TRUE, rsquare = TRUE)

# Extract path coefficients and standard errors

results <- parameterEstimates(fit, standardized = TRUE, ci = TRUE)

results <- results[results$op %in% c("~", ":="), ] # Filter relevant results

# Print the results to check contents and available labels

print(results)

print(unique(results$label))

# Initialize empty vectors for the table

effect_labels <- c("Controlled Direct Effect (CDE)", "Natural Indirect Effect (NIE)", "Total Effect")

estimates <- numeric(length(effect_labels))

standard_errors <- numeric(length(effect_labels))

lower_ci <- numeric(length(effect_labels))

upper_ci <- numeric(length(effect_labels))

p_values <- numeric(length(effect_labels))

# Extract relevant values for each effect

for (i in seq_along(effect_labels)) {

label <- switch(effect_labels[i],

"Controlled Direct Effect (CDE)" = "direct",

"Natural Indirect Effect (NIE)" = "indirect",

"Total Effect" = "total"

)

# Check if the label exists in results

if (label %in% results$label) {

effect_row <- results[results$label == label, ]

if (nrow(effect_row) > 0) {

estimates[i] <- effect_row$est.std[1] # Take the first element if there are multiple

standard_errors[i] <- effect_row$se[1]

lower_ci[i] <- effect_row$ci.lower[1]

upper_ci[i] <- effect_row$ci.upper[1]

p_values[i] <- effect_row$pvalue[1]

} else {

estimates[i] <- NA

standard_errors[i] <- NA

lower_ci[i] <- NA

upper_ci[i] <- NA

p_values[i] <- NA

}

} else {

estimates[i] <- NA

standard_errors[i] <- NA

lower_ci[i] <- NA

upper_ci[i] <- NA

p_values[i] <- NA

}

}

# Create a table to summarize the effects

mediation_table <- data.frame(

Effect = effect_labels,

Estimate = estimates,

Standard_Error = standard_errors,

Lower_CI = lower_ci,

Upper_CI = upper_ci,

p_value = p_values

)

# Display the table

print(mediation_table)
